# Investigation of the combination of intratumoral and peritumoral radiomic signatures for predicting epidermal growth factor receptor mutation in lung adenocarcinoma

**DOI:** 10.1002/acm2.13980

**Published:** 2023-04-01

**Authors:** Yusuke Kawazoe, Takehiro Shiinoki, Koya Fujimoto, Yuki Yuasa, Tsunahiko Hirano, Kazuto Matsunaga, Hidekazu Tanaka

**Affiliations:** ^1^ Department of Radiation Oncology Graduate School of Medicine Yamaguchi University Ube Japan; ^2^ Department of Respiratory Medicine and Infectious Disease Graduate School of Medicine Yamaguchi University Ube Japan

**Keywords:** EGFR mutation, machine learning, radiomic feature

## Abstract

**Purpose:**

We investigated optimal peritumoral size and constructed predictive models for epidermal growth factor receptor (EGFR) mutation.

**Methods:**

A total of 164 patients with lung adenocarcinoma were retrospectively analyzed. Radiomic signatures for the intratumoral region and combinations of intratumoral and peritumoral regions (3, 5, and 7 mm) from computed tomography images were extracted using analysis of variance and least absolute shrinkage. The optimal peritumoral region was determined by radiomics score (rad‐score). Intratumoral radiomic signatures with clinical features (IRS) were used to construct predictive models for EGFR mutation. Combinations of intratumoral and 3, 5, or 7 mm‐peritumoral signatures with clinical features (IPRS3, IPRS5, and IPRS7, respectively) were also used to construct predictive models. Support vector machine (SVM), logistic regression (LR), and LightGBM models with five‐fold cross‐validation were constructed, and the receiver operating characteristics were evaluated. Area under the curve (AUC) of the training and test cohorts values were calculated. Brier scores (BS) and decision curve analysis (DCA) were used to evaluate the predictive models.

**Results:**

The AUC values of the SVM, LR, and LightGBM models derived from IRS were 0.783 (95% confidence interval: 0.602–0.956), 0.789 (0.654–0.927), and 0.735 (0.613–0.958) for training, and 0.791 (0.641–0.920), 0.781 (0.538–0.930), and 0.734 (0.538–0.930) for test cohort, respectively. Rad‐score confirmed that the 3 mm‐peritumoral size was optimal (IPRS3), and AUCs values of SVM, LR, and lightGBM models derived from IPRS3 were 0.831 (0.666–0.984), 0.804 (0.622–0.908), and 0.769 (0.628–0.921) for training and 0.765 (0.644–0.921), 0.783 (0.583–0.921), and 0.796 (0.583–0.949) for test cohort, respectively. The BS and DCA of the LR and LightGBM models derived from IPRS3 were better than those from IRS.

**Conclusion:**

Accordingly, the combination of intratumoral and 3 mm‐peritumoral radiomic signatures may be helpful for predicting EGFR mutations.

## 1 INTRODUCTION

Mutational testing is the standard protocol for determining whether patients with non‐small cell lung cancer (NSCLC) are likely to respond to targeted molecular therapy.^1^ Lung adenocarcinoma is classified as an NSCLC.^2^ Patients with lung adenocarcinoma with epidermal growth factor receptor (EGFR) mutations are treated with EGFR tyrosine kinase inhibitors (EGFR‐TKIs).^3^, ^4^ Treatment with EGFR‐TKIs has given patients better survival rate and longer progression‐free survival times than conventional chemotherapy.^5^ However, the regulation of EGFR mutations by EGFR‐TKIs increases radiosensitivity.^6^ Therefore, identifying the EGFR mutation status is crucial for decision‐making regarding treatment regimens.

Biopsies or surgical specimens are typically obtained for detecting EGFR mutations.^7^ However, these processes are time consuming, expensive, and invasive. Some researchers have proposed predictive models for EGFR mutations using radiomic features derived from computed tomography images.^1^, ^8^, ^9^, ^10^ Radiomics can analyze tumor phenotypes by automatically extracting numerous quantitative features from medical images, such as CT and/or magnetic resonance images.^11^ However, most studies have not considered the radiomic features derived from the peritumoral region and assessed the intratumoral region alone.^7^, ^9^

Some studies have reported the usefulness of radiomic features derived from intratumoral and peritumoral regions for predicting tumor spread in air space (STAS).^12^, ^13^, ^14^ STAS is also associated with EGFR mutations.^14^ Moreover, the predictive model by Wang et al. established that both the intratumoral and peritumoral regions are important for predicting EGFR mutation.^15^

Very few studies have used intratumoral and peritumoral radiomic features to predict EGFR mutation. Yamazaki et al. and Choe et al. reported the usefulness of peritumoral radiomic features in predicting EGFR mutation status.^1^, ^10^ Their methods used a single setting with peritumoral sizes of 3 and 5 mm from the tumor border. Because the studies used different peritumoral size, optimal peritumoral size for predicting EGFR mutation status must be investigated. Therefore, this study explored the radiomic features of the optimal peritumoral size to determine EGFR mutation status and construct machine learning (ML) based predictive models for EGFR mutation status.

## 2 MATERIALS AND METHODS

### 2.1 Patient data

**FIGURE 1:**
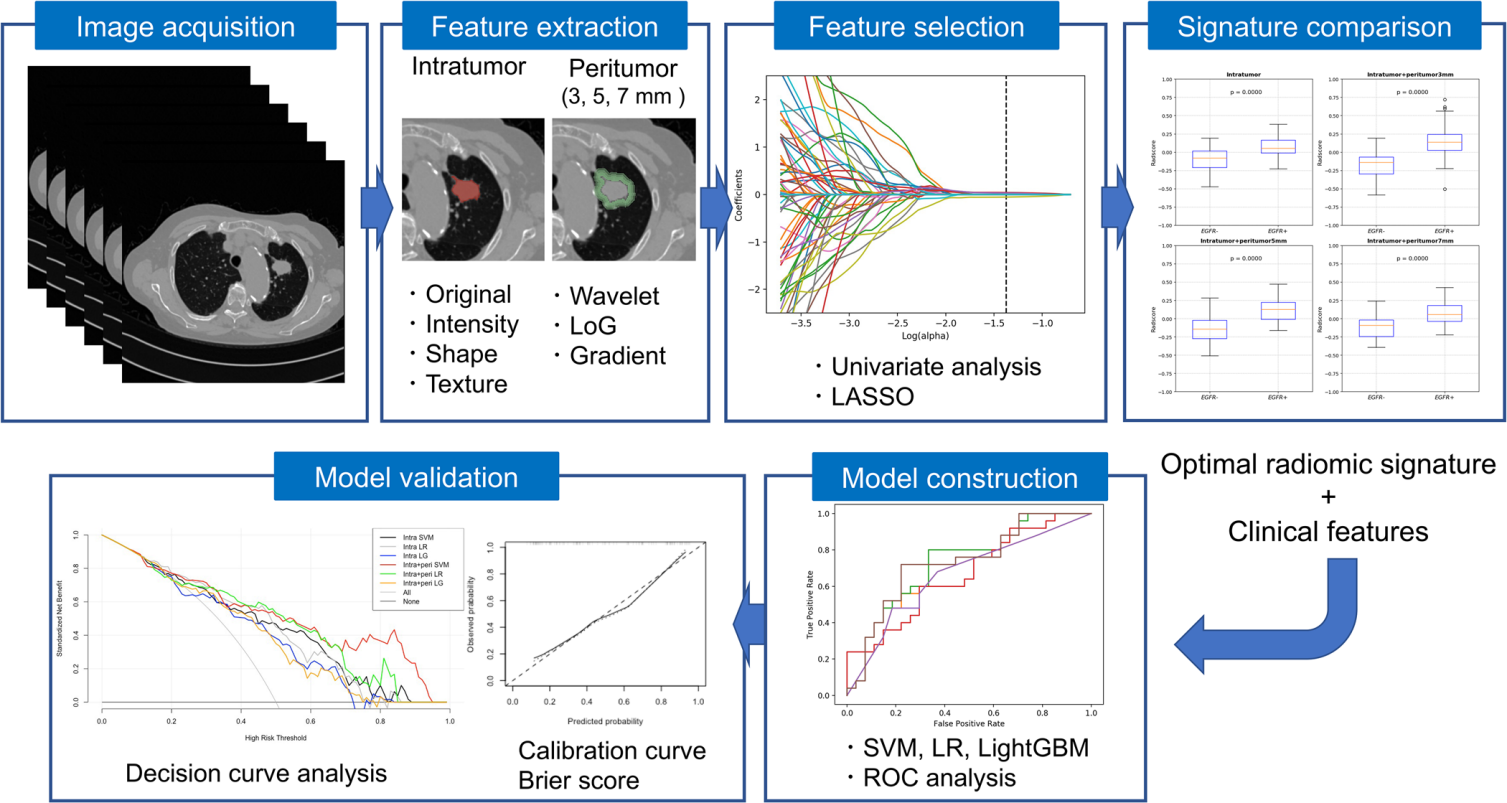
Study design and workflow.

The Institutional Review Board of our institution approved this study. The inclusion criteria were as follows: (a) pathologically confirmed lung adenocarcinoma, (b) confirmed EGFR mutation (EGFR+) or wild‐type (EGFR–), (c) non‐contrast enhanced chest CT images acquired before surgery or targeted molecular therapy or radiation therapy, and (d) only primary tumors. The exclusion criteria were as follows: (a) patients with tumors other than lung adenocarcinoma and, (b) patients who had previously undergone surgery or targeted molecular therapy. A total of 164 patients with NSCLC who had undergone biopsy or surgical specimens between 2016 and 2020 by our institution were randomly selected. These cases were divided into EGFR+ or EGFR– groups in both the training and test cohorts. The data were randomly divided into training and test cohorts with a ratio of 7:3. The clinical features included age, sex, location of the lung tumor, smoking status, and staging. The tumors were divided into five location categories: right upper, right middle, right lower, left upper, and left lower.^2^, ^16^ The detailed characteristics of the patients in our study are shown in Table 1. The study workflow is shown in Figure 1.

**TABLE 1:** Patient characteristics in this study.

Characteristic	Train cohort (*n* = 120)			Test cohort (*n* = 44)		
	EGFR−	EGFR+	*p*‐value	EGFR−	EGFR+	*p*‐value
Age (*y*, mean ± SD)	70.30 ± 9.90	74.15 ± 7.04	0.40	69.30 ± 8.73	67.19 ± 12.07	0.41
Sex, *n*			<0.001			<0.001
Male	45	20		16	8	
Female	15	40		7	13	
Tumor location, *n*			0.25			0.21
Right upper	26	16		11	8	
Middle	0	6		1	1	
Right lower	16	12		4	4	
Left upper	13	17		4	3	
Left lower	5	9		3	5	
Smoking, *n*			<0.001			<0.001
Yes	48	24		21	9	
No	12	36		2	12	
Staging, *n*			0.37			0.37
I	25	33		14	19	
II	5	9		1	1	
III	11	5		4	0	
IV	17	12		4	1	
N/A	3	1		0	0	

Abbreviation: N/A, not available.

### 2.2 CT imaging

CT examinations were performed using five CT scanners: Aquilion Precision (Canon Medical Systems, Otawara, Japan), Optima CT 660 (GE Healthcare, Waukesha, WI, USA), SOMATOM Sensation 64, SOMATOM Force, and SOMATOM Drive (Siemens Healthcare, Forchheim, Germany). The scanning parameters were as follows: tube voltage, 70−120 kV; tube current, automatic exposure control; matrix size, 512 ×512; slice thickness, 1.00 or 1.25 mm; and field of view, 270−400 mm; rotation time of gantry, 0.5 s/rot. All CT images were acquired from patients in the supine position and deep inspiration breath‐hold with both hands raised.

### 2.3 Extraction of radiomic features and feature selection

The acquired images were converted to an isotropic volume (1.00 ×1.00 ×  1.00 mm^3^) using linear interpolation. The intratumoral region of the lung tumor was segmented semi‐automatically using the GrowCut module in the open‐source software 3D Slicer (version 4.10.2, Brigham and Women's Hospital).^17^, ^18^ The pathological usefulness of GrowCut segmentation for NSCLC has been reported.^18^ Two medical physicists observed CT images on the axial, coronal, and sagittal views using the mediastinum (width, 350 HU; level, 40 HU) and lung window (width, 1500 HU; level, −500 HU) settings and performed segmentation. These segmentations were confirmed by a radiation oncologist with over 16 years of experience in radiation therapy. The peritumoral region was determined using quantitative morphologic operations as a radially extending region with 3, 5, and 7 mm radius from the intratumoral region of the tumor boundary.^19^ These peritumoral regions included air in the lungs, pulmonary vessels, and bronchi and did not include the thoracic wall and mediastinum.

Radiomic features were extracted from intratumoral and peritumoral regions using the open‐source software Pyradiomics (version 3.7.1) in Python.^20^ There were 1046 radiomic features extracted from each region, including first‐order (14), shape (18), gray‐level co‐occurrence matrix (GLCM) (22), gray‐level run length matrix (GLRLM) (16), gray‐level size zone matrix (GLSZM) (16), gray‐level dependence matrix (GLDM) (14), Laplacian of Gaussian filters (LoG) (2), gradient filter (1), and wavelet filters (8). The filtered features were acquired by multiplying the above filters by the first‐order, GLCM, GLRLM, GLSZM, and GLDM features. Finally, 1046 radiomic features including first‐order, shape, GLCM, GLRLM, GLSZM, and GLDM features (100) from original image and wavelet (688), LoG (172), and gradient (86) features from filtered image were extracted. The wavelet transform applies a wavelet filter to each CT image, which is then decomposed into low and high frequencies into eight different images.^21^ The major settings for radiomic features extraction were as follows: bin width of feature extraction parameters, 30^22^; sigma size for the LoG filter, 1.0 or 3.0 mm; bin width of the wavelet filter, 10. ResamplePixelSpacing was set to none.^19^ In total, 1046 radiomic features were extracted from intratumoral and peritumoral regions using the above conditions. The combination of intratumoral and peritumoral regions included 2092 radiomic features.

All radiomic features were standardized using the StandardScaler method in the scikit‐learn package.^23^ For the 1046 radiomic features derived from the intratumoral region or 2092 radiomic features derived from the combination of intratumoral and peritumoral regions, the selectKbest method in the scikit‐learn package based on analysis of variance and the least absolute shrinkage and selection operator were applied to training cohorts to reduce redundant features.^9^, ^24^ The *k* value was set to 500 in the selectKbest method. Five‐fold cross‐validation was applied to the training cohort to determine the tuning parameter that regularized the magnitude of the penalization, and features with non‐zero coefficients were selected. The radiomics score (rad‐score) was calculated using a linear combination of selected features multiplied by their coefficients.^9^, ^17^ The rad‐scores calculated from radiomic features derived from intratumoral region and a combination of intratumoral and 3, 5, or 7 mm‐peritumoral regions were evaluated using the Wilcoxon rank‐sum test to determine optimal peritumoral size for distinguishing EGFR+ and EGFR–.

### 2.4 Construction of machine learning based predictive models and performance evaluation

After comparing the rad‐scores, the peritumoral size exhibiting the largest difference between the EGFR+ and EGFR– groups was determined as the optimal peritumoral radiomic signature. Then, combinations of intratumoral and 3, 5, or 7 mm‐peritumoral radiomic signatures were combined with clinical features that showed significant differences, called intratumoral and peritumoral radiomic signatures with clinical features (IPRS3, IPRS5, and IPRS7, respectively). Similarly, we combined intratumoral radiomic signatures and clinical features which showed a significant difference, called intratumoral radiomic signatures with clinical features (IRS). Three ML predictive models (support vector machine [SVM], logistic regression [LR], and LightGBM) were constructed for EGFR mutation status using IRS and PRS. In the SVM model, a radial basis function was applied, and the grid search method with a five‐fold CV was applied to optimize the hyperparameters. In the LightGBM model, to avoid overfitting, it was necessary to add a maximum depth limit; therefore, hyperparameters were optimized using random search in five‐fold CV in the training cohorts.

The predictive performance of each ML model was evaluated using the area under the curve (AUC) of the receiver operating characteristic curve in five‐fold CV. The training models were then evaluated using independent test cohorts. Furthermore, the calibration curve and the Brier score (BS) were used to evaluate the accuracy of ML models, and decision curve analysis (DCA) was used to evaluate the clinical applicability of the ML classifier models.^25^ The BS is calculated by summing the squared difference between the probability of prediction and the real probability.^26^ If the BS is 0, the model is considered to have perfect predictive accuracy; if the BS greater than 0.25, the model is considered to have no value.^25^, ^27^ DCA was performed by calculating the net benefit. The net benefit = true positive rate—(false positive rate × weighting factor), where the weighting factor = the threshold/(1 – threshold). Differences were considered statistically significant at *p* < 0.05. All the procedures were performed using in‐house programs (Python ver. 3.7.1, R ver. 4.1.1).

## 3 RESULTS

**FIGURE 2:**
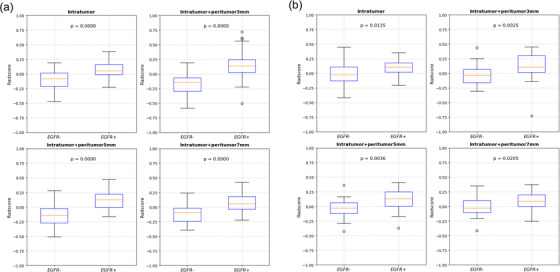
The rad‐score of only intratumoral radiomic signatures and combinations of intratumoral and 3, 5, and 7 mm‐peritumoral radiomic signatures between EGFR+ and EGFR– groups of the (a) training, and (b) test cohorts.

**FIGURE 3:**
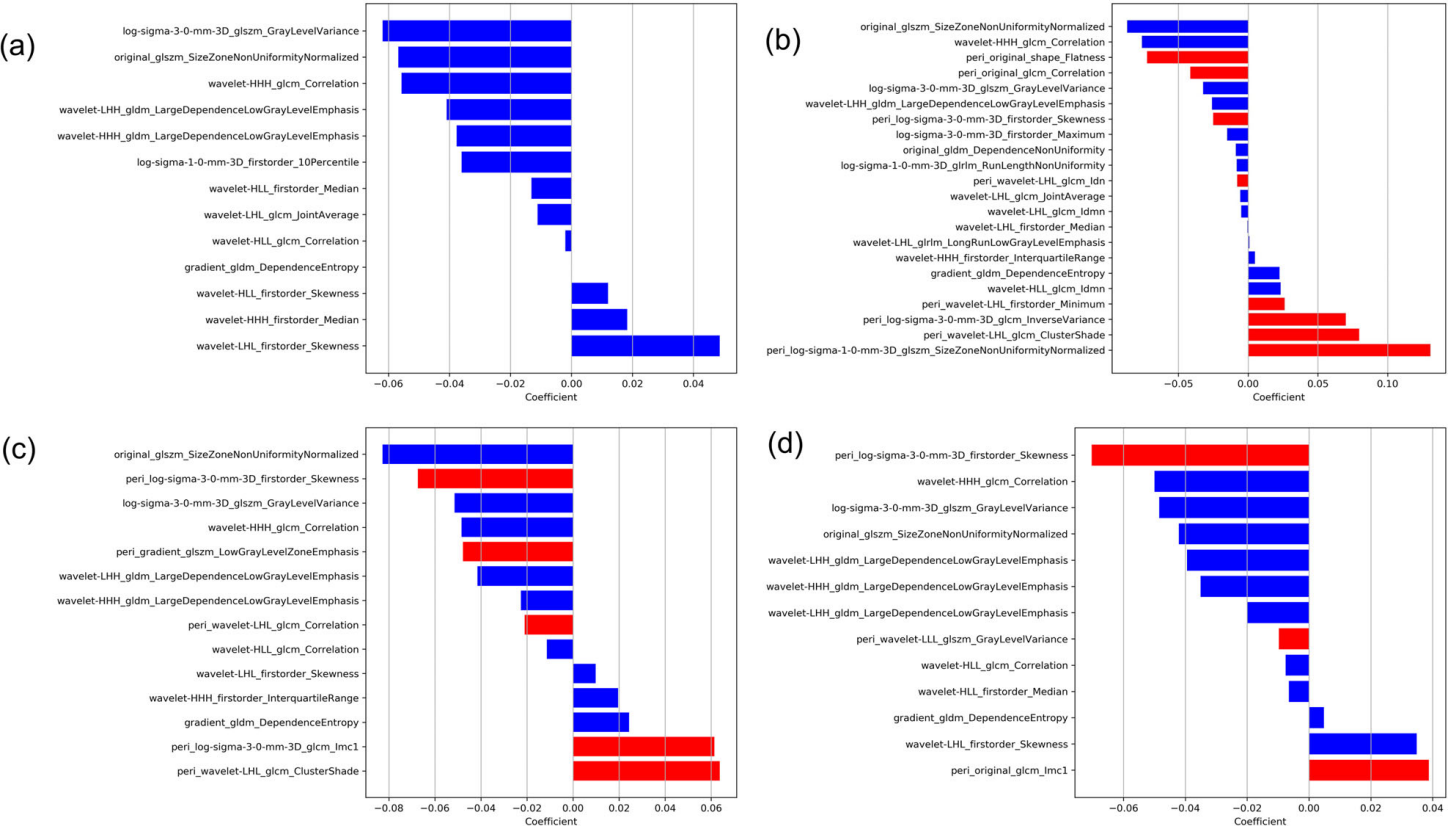
Selected radiomic signatures for calculating the rad‐score of the (a) intratumoral region, (b) combination of intratumoral and 3 mm‐peritumoral regions, (c) combination of intratumoral and 5 mm‐peritumoral regions, and (d) combination of intratumoral and 7 mm‐peritumoral regions.

Among 2092 features derived from combinations of the intratumoral region and 3, 5, and 7 mm‐peritumoral regions, 22, 14, and 13 features were selected, respectively. Among the 1046 features derived from intratumoral features alone, 13 features were selected. Figure 2a shows the rad‐score of the intratumoral radiomic signatures alone and the combinations of intratumoral and 3, 5, and 7 mm‐peritumoral radiomic signatures between the EGFR+ and EGFR– groups for the training, respectively. Figure 2b shows the rad‐scores for the test cohort. The rad‐score showed a significant difference between the EGFR+ and EGFR– groups in the intratumoral and peritumoral regions in both cohorts. In particular, the rad‐score derived from the combination of intratumoral and 3 mm‐peritumoral radiomic signatures showed the largest difference between the EGFR+ and EGFR– groups (training: *p* = 0.0000, test: *p* = 0.0025). Therefore, the optimal peritumoral size was determined to be 3 mm. Figure 3a shows the radiomic signatures for calculating the rad‐score for the intratumoral region, the combinations of intratumoral region and (b) 3 mm‐peritumoral, (c) 5 mm‐peritumoral, and (d) 7 mm‐peritumoral regions.

Differences in clinical features are shown in Table 1, with sex and smoking status being significantly different. Therefore, PRS were constructed using combinations of intratumoral and 3, 5, and 7 mm‐peritumoral radiomic signatures with sex and smoking, namely IPRS3, IPRS5, and IPRS7, respectively, then ML models were constructed using these signatures. Similarly, IRS was constructed using intratumoral radiomic signatures, sex, and smoking status.

Table 2 shows the AUC values for the training and test cohorts for different ML models based on IRS, IPRS3, IPRS5, and IPRS7. For the training cohort, the AUC values in the SVM, LR, and LightGBM models derived from IRS were 0.783 (95% CI:0.602−0.956), 0.789 (0.654−0.927), and 0.735 (0.613−0.958), respectively, and 0.831 (0.666−0.984), 0.804 (0.622−0.908), and 0.769 (0.628−0.921) derived from IPRS3, respectively. For the test cohort, these were 0.791 (95% CI: 0.641−0.920), 0.781 (0.538−0.930), and 0.734 (0.538−0.930) derived from IRS and 0.765 (0.644−0.921), 0.783 (0.583−0.949), and 0.796 (0.583−0.949) derived from IPRS3, respectively.

**TABLE 2:** AUC for training and test cohorts in different ML models based on intratumoral radiomic features with sex and smoking, and the combination of intratumoral and peritumoral radiomic features with sex and smoking.

			Training cohort			Test cohort		
	Classifier	Signature	AUC	[95% CI]	Brier score	AUC	[95% CI]	Brier score
EGFR mutation vs. wild‐type	SVM	IRS	0.783 ± 0.086	[0.602−0.956]	0.189	0.791	[0.641−0.920]	0.196
		IPRS3	0.831 ± 0.059	[0.666−0.984]	0.165	0.765	[0.644−0.921]	0.213
		IPRS5	0.822 ± 0.102	[0.661−0.965]	0.171	0.776	[0.636−0.917]	0.219
		IPRS7	0.715 ± 0.109	[0.215−0.914]	0.219	0.687	[0.529−0.846]	0.233
	LR	IRS	0.789 ± 0.110	[0.650−0.927]	0.189	0.781	[0.538−0.930]	0.207
		IPRS3	0.804 ± 0.068	[0.622−0.908]	0.185	0.783	[0.583−0.949]	0.205
		IPRS5	0.785 ± 0.098	[0.605−0.955]	0.200	0.747	[0.600−0.895]	0.226
		IPRS7	0.779 ± 0.090	[0.597−0.955]	0.215	0.737	[0.588−0.887]	0.233
							
	LightGBM	IRS	0.735 ± 0.091	[0.613−0.958]	0.210	0.734	[0.538−0.930]	0.218
		IPRS3	0.769 ± 0.085	[0.628−0.921]	0.212	0.796	[0.583−0.949]	0.202
		IPRS5	0.736 ± 0.073	[0.537−0.934]	0.219	0.717	[0.537−0.934]	0.216
		IPRS7	0.802 ± 0.071	[0.626−0.973]	0.213	0.755	[0.626−0.973]	0.223

Abbreviations: AUC, area under the receiver operating characteristic curve; CI, confidence interval; EGFR, epidermal growth factor receptor; IPRS3, combination of intratumoral and 3 mm‐peritumoral radiomic signature with clinical features; IPRS5, combination of intratumoral and 5 mm‐peritumoral radiomic signature with clinical features; IPRS7, combination of intratumoral and 7 mm‐peritumoral radiomic signature with clinical features; IRS, intratumoral radiomic signature with clinical features; LR, logistic regression; SVM, support vector machine.

**FIGURE 4:**
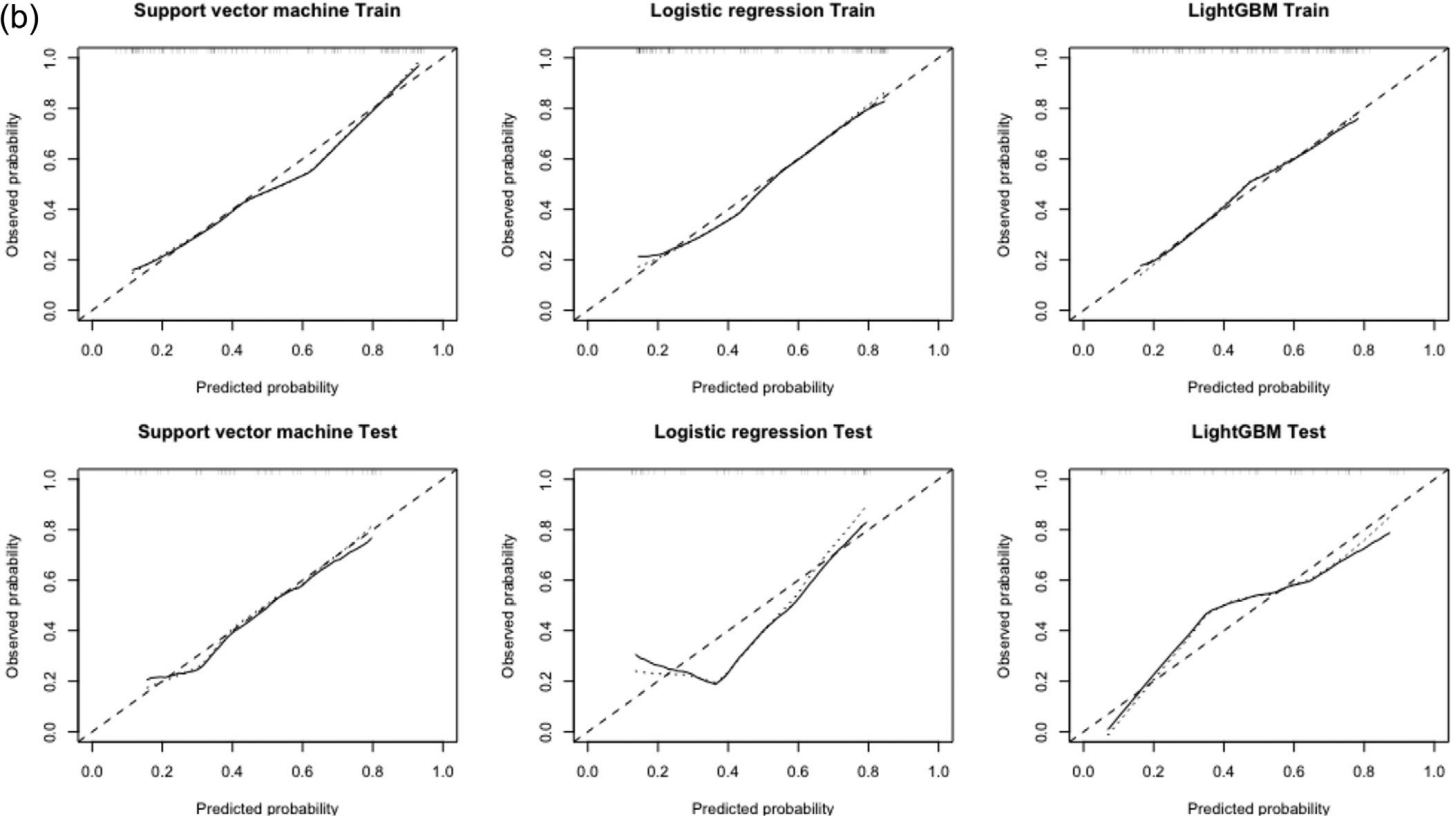
Calibration curves for each machine learning model derived using the combination of intratumoral and 3 mm‐peritumoral radiomic signatures with clinical features. The dashed line shows the ideal model and the solid line shows actual model.

The calibration curves of the predictive models derived from IPRS3 are shown in Figure 4. The calibration curve evaluates the goodness of fit between the predicted probabilities and models with the actual outcomes of EGFR mutation, namely, predictive model accuracy, with the better model being closer to the actual outcome, as shown by the dashed line.^28^ In the training cohort, the goodness of fit between the predicted probability and models with the actual outcomes of EGFR mutations appeared to be good in all models. In the test cohort, the goodness of fit LR and LightGBM models around 0.4 in predicted probability were not well. The BS of the SVM, LR, and LightGBM models in the training cohort were 0.189, 0.189, and 0.210, respectively, derived from IRS, and 0.165, 0.185, and 0.212, respectively, derived from IPRS3. In the test cohort, these were 0.196, 0.207, and 0.218 for IRS and 0.213, 0.205, and 0.202 for IPRS3, respectively.

**FIGURE 5:**
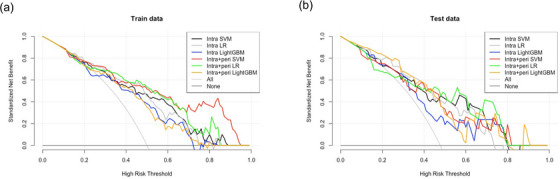
Decision curves of machine learning based predictive models in (a) training and (b) test cohorts.

Figure 5 shows the decision curves of the three ML models for the (a) training and (b) test cohorts. All ML models derived from IPRS3 in the test cohort had more net benefit than “treat all” and the “treat none” with a threshold range over 0.3. Furthermore, in the LR model, compared with IRS, IPRS3 had more benefits in the threshold range from 0.45 to 0.60 and over 0.65 in the test cohort. In the LightGBM model, compared to IRS, IPRS3 in the test cohort had more benefits with the range from 0.05 to 0.55 in the test cohort.

## 4 DISCUSSION

The peritumoral size for determining the EGFR mutation status was optimized. Our results demonstrate that radiomic signatures from the combination of intratumoral and 3 mm‐peritumoral regions could distinguish EGFR+ and EGFR– groups better than 5 or 7 mm‐peritumoral regions. We then constructed predictive models for EGFR mutation status using IPRS3. Our study showed that IPRS3 could better predict EGFR mutation status than IRS.

In terms of clinical features, sex, and smoking status were significantly different (Table 1). Because EGFR mutations are frequently observed in non‐smokers and Asian females, this tendency was reasonable.^3^

As shown in Figure 3, both intratumoral and peritumoral features were selected for all combinations of intratumoral and peritumoral regions. Therefore, it is important to consider the peritumoral region to distinguish the EGFR mutation status, regardless of the size of the peritumoral region. In this study, the optimal peritumoral region was determined to be 3 mm based on the rad‐score results. Our results showed that the ML models derived from IPRS3 performed well. Yamazaki et al. reported the usefulness of 3 mm‐peritumoral radiomic features for predicting EGFR mutation status.^10^ Furthermore, Morales et al. reported that peritumoral lung parenchyma within 3 mm excluding the thoracic wall or mediastinum was correlated with overall survival in lung cancer.^29^ Therefore, the use of IPRS3 is reasonable and meaningful for predicting the EGFR mutation status. However, the optimal peritumoral region can vary depending on the accuracy of intratumoral segmentation. We used GrowCut for tumor segmentation, which segments automatically from a given initial small set of label points in the algorithm.^18^ Therefore, it is expected that the difference in segmentation accuracy due to different operators being used may be reduced. However, because we did not evaluate the reproducibility for segmentation of interclass correlation coefficient, this will be validated.

Previous studies reported developed predictive models for the EGFR mutation status derived from intratumoral radiomic features alone. Zhao et al. reported an AUC value of 0.757 while Mei et al. reported an AUC value of 0.664.^17^, ^30^ Moreover, Choe et al. developed a predictive model using both intratumoral and peritumoral radiomic features for EGFR mutations in lung adenocarcinoma, and the AUC value of their model was 0.64.^1^ In contrast, the AUC value of our best model, LightGBM, demonstrated high performance in the test cohort (0.796). Although validation for a large number of cases is needed, our models derived from IPRS3 may be helpful for predicting EGFR mutation status.

For the calibration curve, all models derived from IPRS3 showed a better goodness of fit in the training cohort. However, in the LR and LightGBM models, the goodness of fit around 0.4 in predicted probability were poor in the test cohort (Figure 4). In the LR and LightGBM models, the BS derived from IPRS3 was slightly better than that derived from IRS. Because a lower BS indicates better model accuracy, these results indicate that the model accuracies of the LR and LightGBM models from IPRS3 are slightly better than that of IRS. Previously, no study has evaluated the model accuracy with BS for EGFR mutation status using 3 mm‐peritumoral radiomic features; therefore, our results are considered to be valuable. However, the validity of these results must be evaluated. Moreover, it has been reported that the predictive model derived from intratumoral radiomic features had a low BS (0.162 in the SVM model)^25^; therefore, the accuracy of our predictive model can be potentially improved.

For the DCA, the LR model derived from IPRS3 had more benefits with the threshold range from 0.45 to 0.60 and over 0.65 than that of IRS in the test cohort. The LightGBM model derived from IPRS3 had more benefits with the threshold range from 0.05 to 0.55 than that of IRS in the test cohort (Figure 5). The threshold is where the expected benefit of treatment and the expected benefit of avoiding treatment are equal.^31^ Moreover, all models derived from IPRS3 showed at least more net benefit than “all treat” or “treat none” with a range over 0.3 in the test cohort. Therefore, clinicians can refer to our results to determine whether the EGFR mutation status based on our models will be useful or not.^32^ According to the results of Liu et al. and Zhang et al., net benefits vary depending on the predictive models.^25^, ^28^ Therefore, validation of several predictive models is important for evaluating the net benefit. Although we validated three ML models, other predictive models need to be investigated.

**FIGURE 6:**
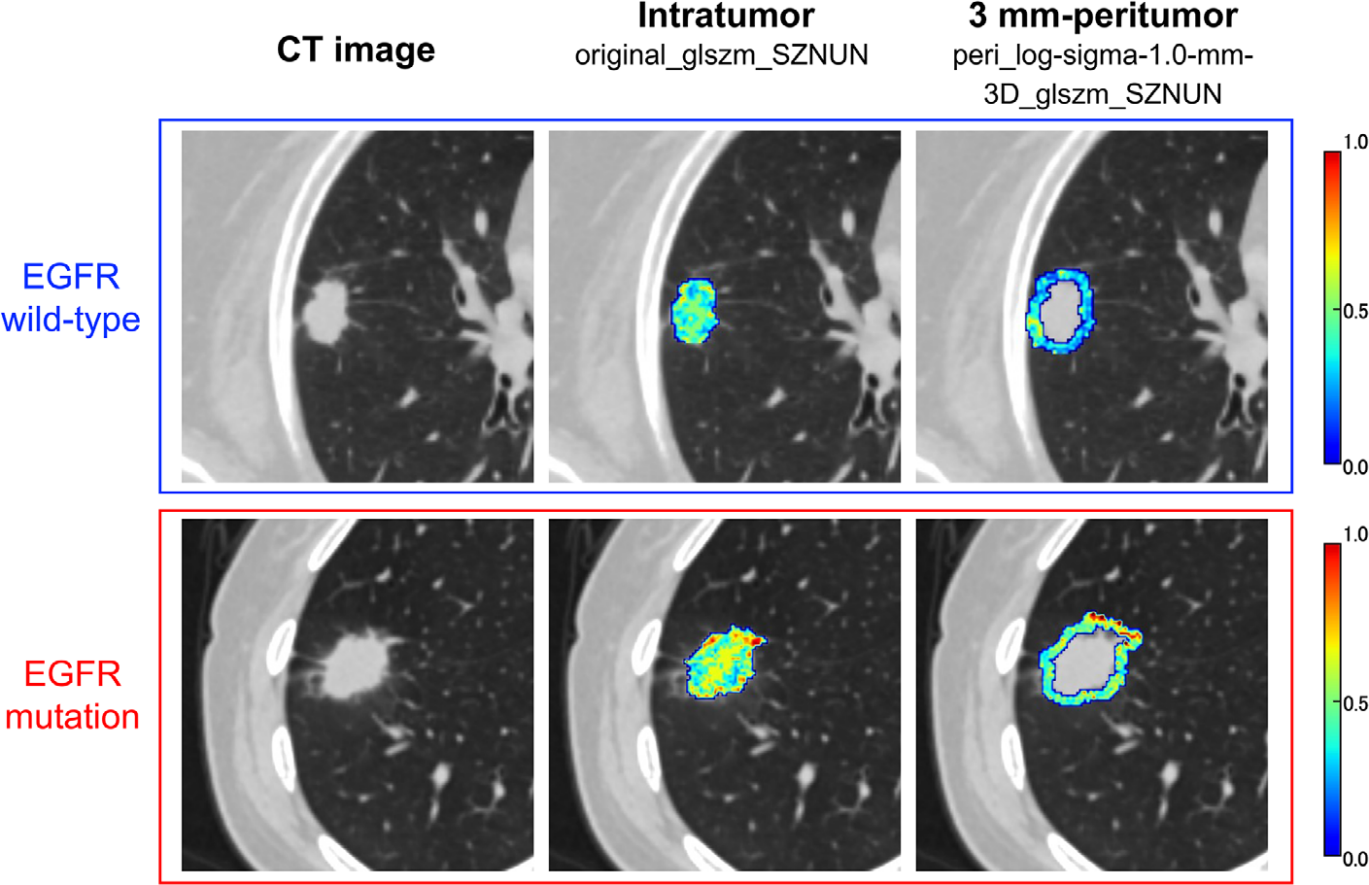
Feature maps generated by glszm_SizeZoneNonUniformityNormalized (center) and peri_log‐sigma‐1‐0‐mm‐3D_glszm_ SizeZoneNonUniformityNormalized (right) in the test cohort with color bar. Feature maps in the EGFR mutation group tended to show high value, whereas those in the wild‐type group showed low value both in intratumoral and peritumoral regions.

The size zone non‐uniformity normalized (SZNUN) feature extracted using GLSZM indicates the variation in volume, and a lower SZNUN indicates greater homogeneity. As shown in Figure 3b, the glszm_SZNUN coefficient was high in both intratumoral (original_glszm_SZNUN: −0.087) and 3 mm‐peritumoral (peri_log‐sigma‐1‐0‐mm‐3D_glszm_ SZNUN: 0.131) features. Examples of the feature maps of these features in the test cohort are shown in Figure 6. The EGFR– and EGFR+ groups demonstrated different tendencies in the feature maps of the original_glszm_SZNUN and peri_log‐sigma‐1‐0‐mm‐3D_glszm_ SZNUN. Biopsy result showing EGFR– can include false negatives because of intratumor heterogeneity.^15^ Therefore, though further evaluation should be performed, the feature map might be helpful for interpreting the heterogeneous areas of the tumor.

An invasive biopsy is required to confirm EGFR mutations patients. Our image‐based method for EGFR mutation identification can eliminate this inconvenient procedure for patients and facilitate early decision‐making regarding treatment strategies. Most studies for predicting EGFR mutation status focused on intratumoral features alone,^8^, ^9^, ^13^, ^16^ while a few studies focused on peritumoral features. Previous studies used a single peritumoral region to construct a single ML model,^1^, ^10^ therefore, the robustness of radiomic signatures in different models is unknown. We compared the radiomic features of multiple peritumoral regions and constructed three ML models. LR and LightGBM models derived from IPRS3 showed similar AUCs and were better than those of IRS, indicating that IPRS3 has high robustness.

Our study has some limitations. First, the number of patients included in this study was limited. Therefore, a larger number of cases should be examined to further validate our results. In addition, we did not validate our predictive models with an external dataset; therefore, it is necessary to compare them with other models. Second, the variety of peritumoral regions was considered insufficient and multiple peritumoral regions to be evaluated in future works. Third, five different CT scanners were used in this study. The variability in the values of radiomics features from different CT scanners can be comparable to the variability in these features in CT images of NSCLC tumors.^33^ Moreover, it is reported that imaging parameters affect the robustness of radiomic features.^34^ Because a lot of facilities have multiple CT scanners, improving robustness of features by imaging parameters correction is necessary. The accuracy of our predictive model may be improved by imaging parameters correction. Furthermore, Zwanenburg et al. reported that image perturbation may be useful for assessing feature robustness.^35^ As future works, we will evaluation feature robustness for extracted radiomic features.

## 5 CONCLUSIONS

We determined the optimal peritumoral size and investigated radiomic features to construct predictive models for EGFR mutation status. The combination of intratumoral and 3 mm‐peritumoral radiomic signatures could identify EGFR mutation status more accurately compared to combinations of 5 or 7 mm‐peritumoral radiomic signatures. Furthermore, LR and LightGBM models derived from IPRS3 demonstrated better accuracy in predicting EGFR mutation status than those derived from IRS. Therefore, the combination of intratumoral and 3 mm‐peritumoral radiomic signatures can help accurately prediction EGFR mutation status.

## AUTHOR CONTRIBUTIONS

Yusuke Kawazoe and Takehiro Shiinoki designed this study. Yusuke Kawazoe and Koya Fujimoto carried out the experiment. Yusuke Kawazoe wrote the manuscript with support from Takehiro Shiinoki. Koya Fujimoto, Yuki Yuasa, Tsunahiko Hirano, Kazuto Matsunaga, and Hidekazu Tanaka supplied available data in terms of this study and discussed. All authors discussed the results and contributed to the final manuscript.

## CONFLICT OF INTEREST STATEMENT

The authors declare no conflicts of interest.

## Data Availability

The data that support the findings of this study are available from the corresponding author upon reasonable request.
